# Efficacy of Cognitive Behavioral Therapy on Opiate Use and Retention in Methadone Maintenance Treatment in China: A Randomised Trial

**DOI:** 10.1371/journal.pone.0127598

**Published:** 2015-06-24

**Authors:** Shujun Pan, Haifeng Jiang, Jiang Du, Hanhui Chen, Zhibin Li, Walter Ling, Min Zhao

**Affiliations:** 1 Shanghai Mental Health Center, Shanghai JiaoTong University School of Medicine, Shanghai, China, 200030; 2 Integrated Substance Abuse Programs, Department of Psychiatry and Biobehavioral Sciences, David Geffen School of Medicine, University of California Los Angeles, Los Angeles, California, United States of America; Centre for Addiction and Mental Health, CANADA

## Abstract

**Aims:**

Methadone maintenance treatment (MMT) is widely available in China; but, high rates of illicit opiate use and dropout are problematic. The aim of this study was to test whether cognitive behavioral therapy (CBT) in conjunction with MMT can improve treatment retention and reduce opiate use.

**Method:**

A total of 240 opiate-dependent patients in community-based MMT clinics were randomly assigned to either weekly CBT plus standard MMT (CBT group, n=120) or standard MMT (control group, n=120) for 26 weeks. The primary outcomes were treatment retention and opiate-negative urine test results at 12 weeks and 26 weeks. The secondary outcomes were composite scores on the Addiction Severity Index (ASI) and total scores on the Perceived Stress Scale (PSS) at 12 weeks and 26 weeks.

**Results:**

Compared to the control group in standard MMT, the CBT group had higher proportion of opiate-negative urine tests at both 12 weeks (59% vs. 69%, p<0.05) and 26 weeks (63% vs. 73%, p<0.05); however, the retention rates at 12 weeks (73.3% vs. 74.2%, p=0.88) and 26 weeks were not different (55.8% vs. 64.2%, p=0.19) between the two groups. At both 12 and 26 weeks, all of the ASI component scores and PSS total scores in the CBT group and control group decreased from baseline; but the CBT group exhibited more decreases in ASI employment scores at week 26 and more decrease in the PSS total score at week 12 and week 26.

**Conclusions:**

CBT counselling is effective in reducing opiate use and improving employment function and in decreasing stress level for opiate-dependent patients in MMT in China.

**Trial Registration:**

ClinicalTrials.gov NCT01144390

## Introduction

Drug abuse has been prevalent in China for decades. In 2013, the number of registered drug users on the Chinese mainland reached 2.2 million [1], with heroin being the most commonly used illicit drug. Heroin and related problems have become a major concern for the Chinese government and for public health departments. The prevalence of HIV, HCV and co-infection with both was 6.0%, 60.1%, and 4.6%, respectively, among heroin-dependent people in methadone maintenance treatment (MMT) programs on the Chinese mainland [2]. Approximately 780,000 people are HIV seropositive and receive treatment for AIDS in China [3]. MMT was introduced in 2004 to respond to the problems of heroin and to reduce consequences, and there are more than 700 community-based MMT clinics in China [4]. However, lapse, relapse, and premature dropout among MMT patients are commonly reported [5,6]. Findings from studies exploring the reasons for poor outcomes of MMT have suggested that the majority of these patients had various psychological problems and emotional disorders, held negative attitudes toward MMT, and had various difficulties and stresses associated with abstinence during the recovery process [7,8,9]. These problems are barriers to treatment outcomes and must be addressed in MMT programs. One strategy is to combine a psychosocial intervention to improve treatment efficacy. However, formal psychological or behavioral interventions are not implemented in most MMT programs in China for various reasons, such as personnel shortage and resource limitations.

For decades, cognitive behavioral therapy (CBT), which combines cognitive and behavioral theory, has been one of the most applied behavioral interventions for patients with different mental health disorders [10,11,12]. CBT alone or combined with pharmacological treatments has been used for substance use disorders (SUDs) [13,14], as well as for other mental health co-mobidities [15,16,17,18,19]. CBT for SUDs focuses on re-constructing distorted cognition regarding patients themselves, others, and the environment, engaging in skills training in coping, and rebuilding a balanced lifestyle for sustaining abstinence [13,20,21]. Researchers have suggested that more CBT sessions would achieve better performance and longer periods of abstinence for chronic substance users, although there is considerable variability in CBT sessions. The format of CBT for SUDs was either individual [22] or group sessions [23]; individual sessions focused on individualized treatment plans, whereas group sessions focused on sharing experiences and peer-support for sustaining abstinence.

Evidence from some clinical studies has shown that CBT is effective for patients with SUDs [24,25], but the literature is inconsistent concerning the efficacy of CBT for MMT patients. Several studies have supported that CBT had long-lasting efficacy on reducing cocaine use at post-treatment follow-up for patients in MMT who also used cocaine [26,27,28]. However, other studies do not yield findings of better treatment outcomes of CBT for MMT patients [29]. There are no research studies on the use of CBT for efficacy on improving the outcomes among Chinese MMT patients. Considering the existing problems among Chinese MMT patients, we implemented CBT to address psychological and cognitive problems related to relapse and premature dropout to improve treatment outcomes. The present study aimed to examine (1) whether CBT is effective in improving treatment retention and reducing drug use for opiate-dependent Chinese patients in MMT and (2) whether CBT is effective in decreasing addiction severity and psychological stress for MMT patients.

## Participants and Method

### Study Participants

**Fig 1:**
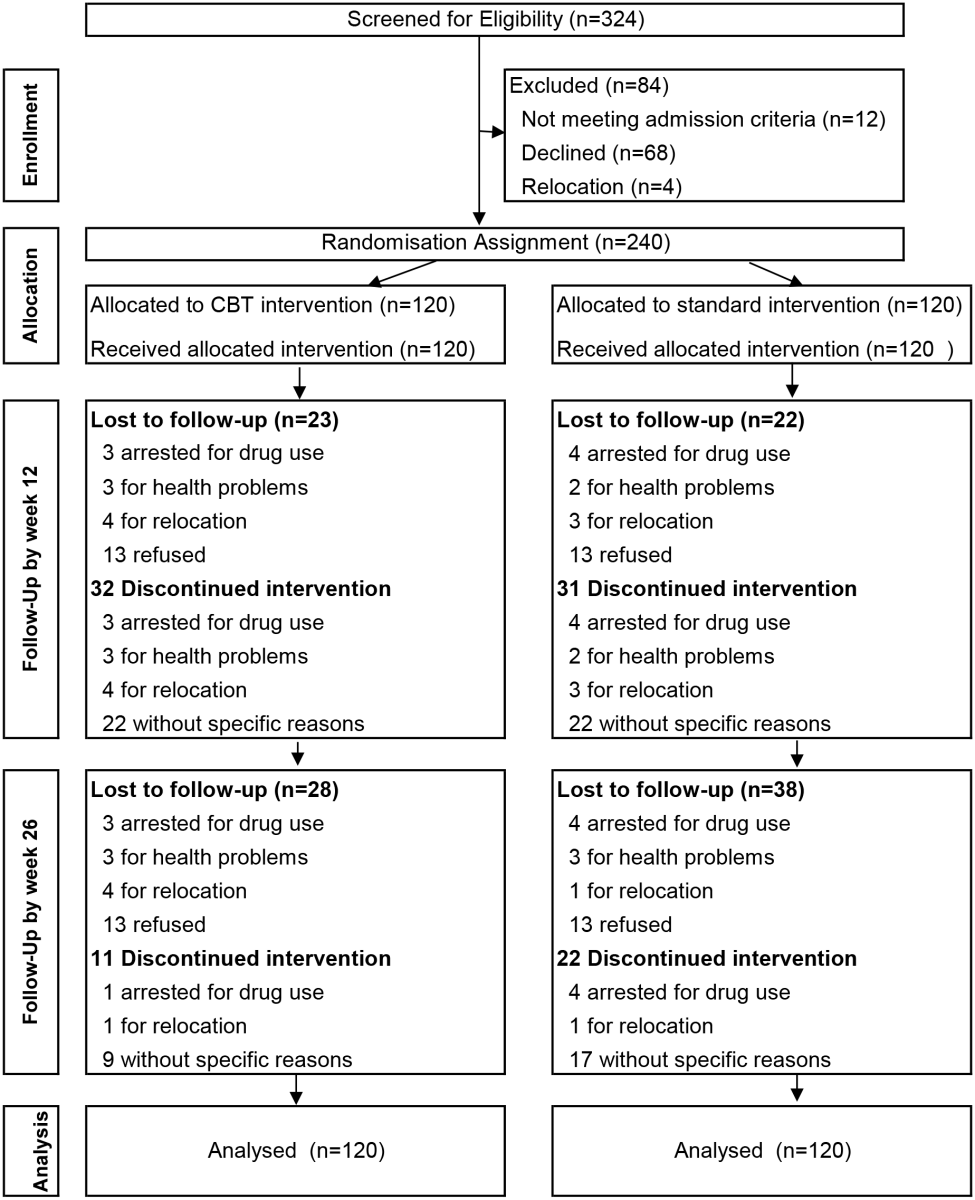
Flowchart of participants.

The sample size was determined using NCSS PASS v11.0 (Kaysville, UT), on the basis of a 60% retention rate estimated from our previous study and clinical data, with the statistical power at 80% and the significance level at 0.05. An increase of 15% in retention rates due to implementation of CBT was also expected. Thus, about 120 cases were needed in each group for a one-side test; total of 240 participants were enrolled in the study. Consecutive admissions to the four participating community-based MMT clinics in Shanghai occurred from April 2010 to June 2011. Participants were included in the study if they met DSM-IV criteria for opiate dependence diagnosed by psychiatrists in the MMT clinics; they were age 18 to 65 years, had no serious mental and physical disorders, and were not participating in any other study. Overall, of 324 individuals invited, 68 patients declined to participate in the study, 12 patients were excluded because they did not fulfill the study inclusion criteria, and four patients were excluded because they had relocated. A total of 240 individuals agreed to participate in the study and provided their signed informed consent before randomization. Using a computer-generated randomization sheet, the participants were randomly assigned to either the CBT group (n = 120) or the control group (n = 120) (Fig 1). The participants were stratified according to gender, age, and years of opiate use before randomization. The clinical directors of the MMT clinics were the site principal investigators in charge of the randomization assignment using a sheet of assignment numbers that was locked in the drawer. Counselors administered both CBT and standard care for participants assigned to him/her. The ASI and PSS were conducted by independent trained interviewers who did not know the group assignment.

The study was approved by the Institutional Review Boards of Shanghai Mental Health Centre (SMHC IRB: 2009036) in December 2009 and registered at ClinicalTrials.gov (registration number NCT01144390) in June 2010. The protocol for this trial and supporting CONSORT checklist are available as supporting information; see S1 CONSORT Checklist, S1 Protocol (English Version) and S1 Protocol (Chinese Version).

### Standard Care Treatment

The participants in both groups received standard MMT for 26 weeks. This protocol involved gradually increasing the methadone dose in the first week to a level that controlled the craving for drugs. In addition to daily oral administration of methadone, the participants received monthly health education in the form of lecture and voluntary counselling and testing for HIV. The participants in the control group received only standard MMT. Regular attendance in MMT clinics means attending MMT clinics to take daily oral methadone under staff supervision. Participants were permitted to have gaps of no more than consecutive 7 days with appropriate excuses in the study period.

### Cognitive Behavioral Therapy Procedure

The participants in the CBT group received individual CBT weekly and group CBT monthly in addition to the standard care of MMT treatment for 26 weeks. The CBT was delivered by psychotherapists experienced in providing counselling or psychotherapy services for patients with SUDs and mental health disorders. They received training for the study in a 3-day didactic and interactive seminar. The competence of CBT counselling was rated with the validated rating system after training. The CBT manual used for this study was adapted from the Matrix Intensive Outpatient Treatment for People With Stimulant Use Disorders [30] by the study group in collaboration with colleagues at the Integrated Substance Abuse Programs (ISAP) at UCLA.

The individual CBT sessions occurred in three stages over 6 months. The focus of the first stage of individual sessions (first 6 weeks) was to build treatment relationships and enhance motivation for MMT. By a comprehensive evaluation of the patient’s opiate use history and drug-related problems, the patients understood their physical, mental health, social function, legal, economic, family, and employment problems associated with their opiate use. In this stage, the principle of CBT was explained and the participants signed a treatment agreement and set treatment goals with their counselors. The second stage (from week 7 to week 14) focused on skills training in coping and in recognition and management of triggering and craving for opiate use. The goal of the second stage was to make individualised intervention protocols, proceed step by step according to the individualised protocols, and repeatedly evaluate treatment feedback. Considering the feedback from previous sessions, the counselors focused on problem solving and skills building. The third stage (from week 15 to week 26) focused on managing psychological stress, building a balanced lifestyle, and maintaining abstinence. CBT group sessions addressed health education, recognition and self-control of drug craving, harm reduction, and relapse management. The participants in CBT group sessions shared with other group members their experiences and knowledge of ceasing drug use and maintaining abstinence.

### Treatment Fidelity

To avoid therapist-by-condition confounding, each therapist provided either CBT or standard care to an equivalent number of participants in each group. To ensure the fidelity of CBT sessions administered by therapists, quality control for the entire study protocol was undertaken by supervisors from the central site. Using individual case analysis or group discussions, licensed senior therapists with experience in CBT provided supervision for counselors to ensure that CBT was administered according to the proper guidelines. This approach was used because evidence indicates that individual feedback and coaching are more likely to enhance skills in delivering behavioral interventions [31]. Group supervision of the CBT intervention were recorded and discussed by the study group.

### Assessment and Outcome Measurement

The primary outcome of the study was retention in treatment and opiate use during the study period. The secondary outcomes of the study were composite scores of the ASI and PSS total scores. Participants were assessed at baseline, week 12, and week 26. The methadone dose was recorded in accordance with the National MMT data management system.

#### Treatment Retention

Treatment retention was defined as regular attendance for methadone medication in MMT clinics. Participants would be discharged from the study if they did not attend MMT clinics to take methadone for seven consecutive days.

#### Drug Use

Opiate use was measured by urine samples. Study research assistants collected urine samples 2 times per month. All of the urine specimens were collected on-site under staff observation and immediately screened for opiates. Consistent with other studies and our previous observations, almost all patients who failed to provide urine for test or were lost to follow-up had relapsed. Therefore, any missing urine specimens were assumed positive, and the proportion of negative urine tests was defined as the proportion of the total number of negative drug urine tests among the total required number of drug tests during a certain time periods (for example, 6 times at 12 weeks and 13 at 26 weeks were the required numbers of drug tests). The overall proportion of negative urine test results was used to determine the opiate use status.

#### Addiction Severity

The addiction severity of patients was measured at baseline, week 12, and week 26 using the Chinese version of the ASI. The ASI was developed [32] in the 1980s and was later translated into Chinese. The Chinese version [33] has exhibited good validity and reliability for opiate-dependent Chinese patients. Cronbach’s alpha coefficient ranged between 0.44–0.79 and the test-retest reliability ranged between 0.68–0.84 for Chinese samples from MMT clinics. The participants were assessed in seven domains of the ASI: physical health, mental health, employment, family support, opiate use, alcohol use, and legal problems. The range of the composite ASI score was between 0 and 1; the higher scores indicated more serious problems.

#### Stress level

The Perceived Stress Scale (PSS) was administered at baseline, week 12, and week 26. The PSS contained 10 items and was used to evaluate psychological stress during the last 30 days. A higher score means a higher level of psychological stress. The PSS was designed [34] and translated into Chinese and tested for validity and reliability for Chinese samples. The Chinese version of the PSS [35] demonstrated good reliability with 0.86 for Cronbach’s alpha coefficient, and 0.68 for re-test reliability coefficient in Chinese female police.

### Data Analysis

The primary endpoint of this study is the retention rate at 26 weeks, and the secondary endpoints included retention rate at 12 week, ASI and PSS scores at 12 and 26 week. Baseline characteristics were assessed using analyses of variance (ANOVA) for continuous variables and chi-squared tests for dichotomous variables. Mann-Whitney tests were used for analysis of non-normally distributed variables (seven composite ASI scores). A Kaplan-Meier survival analysis with ‘dropout’ as the outcome measure (defined as seven consecutive days without methadone) was used to compare the length of stay in MMT program between two groups at 26 weeks. Longitudinal repeated-measure data of ASI and PSS from baseline, week 12 and week 26 assessment points were analyzed using generalized estimating equation (GEE). A few variables on drug use history (drug use methods, rehabilitation history) for 3 participants and employment status for 2 participants were missing. An intent-to-treat model was used for the analysis of ASI and PSS results, hence included all subjects who were randomized and received at least one session of the 26 week study. Independent t test was used to compare changes on ASI employment scores and PSS total scores from baseline to week 12 and week 26 assessment points.

## Results

### Demographic Characteristics and Baseline Status of MMT Patients

The mean age of participants was 40.9 (SD = 8.5) years, 77.5% were male, 35% were married, 62.9% had less than a high school diploma, 70.4% had been unemployed in the past three years and 12.6% of the participants had chronic physical illnesses. The average age at the time of first opiate use was 29.2 (SD = 8.6) years, and the mean duration of opiate use was 9.5 (SD = 4.5) years; 67.9% of participants were opiate injectors. There were no significant group differences in the baseline characteristics (Table 1). The employment score was highest among the ASI composite scores (mean = 0.57, SD = 0.26), followed by family, drug, physical, and mental health components (Table 2), with no significant differences in seven ASI composite scores between groups. The average PSS total score was higher in the CBT group than in the control group at baseline (CBT: mean = 19.67, SD = 4.23; control group: mean = 18.46, SD = 4.59, *p*<0.05).

**Table 1:** Baseline characteristics of participants.

	CBT Group (n = 120)	Control Group (n = 120)	
Mean age, M (SD), year	40.9 (8.0)	40.9 (9.0)	0.981
Male, n (%)	93 (77.5%)	93 (77.5%)	NA
Married, n (%)	44 (36.7%)	40 (33.3%)	0.780
^Education level, M (SD), year	9.9 (1.9)	9.9 (1.8)	0.784
Less than high school, n (%)	69 (57.5%)	82 (68.3%)	0.109
Unemployed in last 3 years, n (%)	90 (75.0%)	79 (65.8%)	0.157
Chronic physical illness, n (%)	16 (13.3%)	14 (11.7%)	0.846
Age of first use, M (SD), year	29.3 (8.4)	29.0(8.8)	0.776
Years of use, M (SD), year	9.8 (4.8)	9.1 (4.2)	0.205
*Life-time injection, n (%)	85 (70.8%)	78 (65.0%)	0.407
*Mandatory rehabilitation history, n (%)	90 (75.0%)	78 (65.0%)	0.121
Re-education through labour, n (%)	61 (50.8%)	66 (55.0%)	0.605

CBT, cognitive behavioral therapy; M, mean; SD, standard deviation.

^^^ There are two subjects from CBT group with missing data.

* There are three subjects from CBT group with missing data.

**Table 2:** ASI composite scores and the PSS at baseline and week 12 and 26, M (SD).

				Statistical analysis GEE			
Dimension	Time*	CBT Group	Control Group	Main effects of group: *p value*	Main effects of time: *p value*	Interaction of group and time: *p value*	Goodness of Fit (Model): QIC* value
Physical	Baseline	0.14 (0.28)	0.11 (0.22)				
Health	Week 12	0.07 (0.22)	0.13 (0.34)				
	Week 26	0.05 (0.14)	0.05 (0.17)	0.739	0.001	0.202	46.712
Employment	Baseline	0.59 (0.24)	0.56 (0.27)				
	Week 12	0.49 (0.27)	0.54 (0.25)				
	Week 26	0.45 (0.27)	0.55 (0.24)	0.104	0.022	**0.032**	52.106
Alcohol Use	Baseline	0.05 (0.12)	0.05 (0.10)				
	Week 12	0.04 (0.08)	0.03 (0.05)				
	Week 26	0.02 (0.05)	0.03 (0.06)	0.932	0.001	0.072	15.706
Opiate use	Baseline	0.18 (0.07)	0.18 (0.07)				
	Week 12	0.04 (0.05)	0.05 (0.05)				
	Week 26	0.04 (0.06)	0.05 (0.06)	0.401	0.001	0.549	14.065
Illegal	Baseline	0.09 (0.17)	0.11(0.19)				
Problems	Week 12	0.02 (0.05)	0.01 (0.04)				
	Week 26	0.01 (0.02)	0.01 (0.04)	0.632	0.001	0.556	18.554
Family	Baseline	0.33 (0.39)	0.41 (0.45)				
Support	Week 12	0.28 (0.30)	0.27 (0.31)				
	Week 26	0.30 (0.34)	0.25 (0.28)	0.778	0.001	0.111	88.665
Mental Health	Baseline	0.13 (0.20)	0.12 (0.19)				
	Week 12	0.04 (0.08)	0.04 (0.11)				
	Week 26	0.05 (0.12)	0.04 (0.11)	0.880	0.001	0.843	23.910
Psychological Stress	Baseline	19.67 (4.23)	18.46 (4.59)				
	Week 12	16.30 (5.02)	17.04 (5.28)				
	Week 26	15.51 (5.02)	17.13 (5.12)	0.402	0.001	**0.008**	14257.767

CBT, cognitive behavioral group; M, mean; SD, standard deviation. All data were presented as M(SD).

* The sample size was 97 for CBT group and 98 for control group at week 12, 92 for CBT group and 82 for control group at week 26.

### Retention in Treatment and Drug Use

Table 3 shows the retention status, retention length, urine drug test results and methadone dosages at week 12 and week 26. Although participants at week 26 had a higher retention rate in the CBT group than the control group, the difference was not statistically significant. The average proportion of opiate-negative urine samples in the CBT group was higher than that in the control group at week 12 (t = 6.02, *p* = 0.02) and week 26 (t = 5.81, *p* = 0.02). The average days stay in MMT (CBT group: mean = 140.29, standard error of the mean = 5.50; control group: mean = 133.65, standard error of the mean = 5.69; log-rank test, χ² = 1.57, *p* = 0.21) and mean dosage (mg/day) of methadone (CBT: mean = 51.04, SD = 21.73; control group: mean = 46.17, SD = 21.02; t = 3.12, *p* = 0.08) did not differ significantly between two groups.

**Table 3:** Retention rates and opiate use for two groups.

	CBT Group	Control Group	Total	T / χ²	*p*
Retention rate, n(%)					
at week 12	88(73.3%)	89(74.2%)	177(73.8%)	0.02	0.88
at week 26	77(64.2%)	67(55.8%)	144(60.0%)	1.74	0.19
Average proportion of negative urine samples, mean(SD) (n = 120)					
at week 12	0.69(0.28)	0.59(0.35)	0.64(0.32)	6.02	0.02
at week 26	0.73(0.29)	0.63(0.37)	0.68(0.34)	5.81	0.02

CBT: cognitive behavioral therapy.

### Addiction Severity and Stress Level

Among 240 participants, 63 participants dropped out of the study by week 12; 32 participants were in the CBT group and 31 participants were in the control group (Fig 1). Of these 63 participants, 18 were located and completed the 12-week follow-up interview (9 were in the CBT group and 9 were in the control group). In total, 81.25% (195/240) of participants completed an interview at week 12 (97 were in the CBT group, 98 were in the control group). An additional 33 participants had dropped out from the study at week 26. In total, 72.5% (174/240) completed the follow-up interview at week 26 (92 were in the CBT group, 82 were in the control group). There was no significant difference between the groups in the proportion of participants who completed the interviews at either week 12 or week 26.

ASI composite scores and PSS total score are presented in Table 3. The results of the GEE analysis showed that the effect of time was significant for all of the ASI component scores and PSS total score; however, the effect of the group was not significant. It indicated that the average scores for each component of the ASI declined significantly from intake to week 12 and week 26 (*p*<0.05) for both groups; there were no differences for any of the ASI component scores and PSS total score between the two groups. The GEE analysis showed that there was a significant interaction effect between time and group in the employment component score (*p*<0.05) and PSS total score *(p*<0.05), which indicated that the changes of employment component score and PSS total score were different between the two groups. Therefore, we examined the differences of score changes of employment component score and PSS total score at week12 and week 26 from the baseline between the two groups. The results showed the CBT group had more reductions in the employment composite score of the ASI from baseline to week 26 (CBT group: mean = 0.13, SD = 0.35; control group: mean = -0.01, SD = 0.36, t = -2.24, *p* = 0.025) and CBT participants also had more reductions in the PSS total score from baseline to week 12 (CBT group: mean = 3.34, SD = 5.97; control group: mean = 1.15, SD = 6.88, t = -2.27, *p* = 0.02) and week 26 follow-up (CBT group: mean = 3.91, SD = 5.92; control group: mean = 0.68, SD = 6.61, t = -2.90, *p* = 0.004). Analyses revealed that the CBT groups improved more on employment function at 26 weeks, and decreased more on stress level at both week 12 and week 26 compared with the control group.

## Discussion

To the best of our knowledge, this report is the first study on the efficacy of CBT among MMT patients in China. The primary aim was to examine the efficacy of a manual-based CBT intervention for MMT patients to improve treatment retention, reduce opiate use, and reduce addiction severity and psychological stress compared to standard MMT without CBT. The primary analysis revealed that retention rate at week 26 from participants in the MMT combined with CBT group was not significantly different than those from the MMT-only group. Some previous studies [26,27,28] also did not find a significant effect of CBT during treatment, however, findings from follow-up suggested that delayed effects may occur after the cessation of short-term CBT. It was necessary to follow-up the participants for a period after the intervention stopped to explore whether CBT would produce comparable long-term outcomes. We observed that participants who received the CBT intervention had a higher proportion of negative urine test results for opiates at week 12 and week 26. This finding is consistent with other studies, which showed that CBT is effective in reducing opiate use and relapse [18,23,36,37,38].

There was no significant improvement in retention rates and retention length for patients in the CBT group, although the patients in the CBT group tended to have higher retention rates than the control group (64.2% vs.55.8%) during 26 weeks after entering into the MMT program. The difference in retention rates may be attributed to the following reasons: CBT for drug users was essentially designed to reduce drug use and relapse; therefore, the primary effect was to reduce drug use. There are many factors related to MMT retention, such as the patient’s characteristics, psychosocial factors, medication dosages, and other variables related to treatment [9,39,40,41]. CBT primarily addressed psychological and environmental high-risk situations related to drug use, but CBT did not specifically address the factors related to retention; therefore, we did not find that CBT had an effect on improving retention rates in the study. When implementing CBT for MMT patients, CBT should be modified to address specific factors related to treatment retention. Other factors related to retention such as increase dosages of methadone should be considered in future interventions for improving MMT retention. Because we observed that the average dosage of methadone (48.6 mg/day) in the study was much lower than the medication level (more than 80 mg/day) that was suggested for patients to be retained in MMT [42]. Lastly, the sample size was estimated based on the expected improvement on retention rates of 15%, future study is needed to confirm effects of CBT on retention in a larger sample.

We also examined whether CBT is effective for decreasing the severity of addiction and psychological stress. The baseline ASI scores demonstrated that the participants had problems in several areas, in which the most impaired areas were employment and family function. The PSS total score at baseline demonstrated that the participants had a high level of psychological stress. These problems were similar to other patients in MMT in China [4,6,43,44]. The problems can be both consequences of chronic drug use and barriers for recovery, which should be addressed in the treatment process [5,9,45]. We found that all of the ASI component scores and the PSS total score in both CBT and control groups at 12 weeks and 26 weeks were decreased from baseline, which confirmed that MMT is effective in reducing addiction severity and psychological stress as shown in previous studies [5,7,8,40]. Additionally, the findings indicated that the CBT group had more improvements on ASI employment scores and the PSS total score than the control group. The findings demonstrated that CBT, which involves skills training in coping and relapse prevention for dealing with drug-related circumstances, is effective in improving employment function and reducing psychological stress. The results were supported by other studies on the efficacy of CBT for substance abuse populations. For example, one study showed that substance use was reduced and that occupational function was improved significantly through a 20-session CBT intervention by the end of treatment [17]. Another study that compared CBT with a 12-step facilitation therapy in a pharmacotherapy platform showed that the CBT intervention had significant efficacy on reducing substance use and depression post treatment [46].

The ASI and PSS measures do not include data on those who had severe relapses so it underestimated the true severity of the abuse in the subjects. Since the number of dropouts increased over the three assessment periods (baseline, 12 weeks and 26 weeks) it is possible that some of the reported improvement in the severity measures of abuse over time are cause by the removal of the most severely affected individuals. However the proportion of participants who completed ASI and PSS assessment was relatively acceptable (about 70%), and the proportions of participants who completed the interviews at either week 12 or week 26 in the two groups was similar. It therefore seems unlikely that the dropouts lead to biased results.

### Limitations

This report is the first study to adapt and implement a manual-based CBT intervention and to evaluate its efficacy for MMT patients in China. There are several limitations that should be considered. First, the frequency of collecting urine samples (once every two weeks) may not be sufficient to detect all likely incidents of drug use. Although urine samples were collected at random and the patients were not informed in advance of the testing, the less frequent testing may underestimate drug use in MMT patients. More frequent collection of urine samples is recommended in future studies. Second, more time may be needed for MMT patients to incorporate the skills learned in CBT and to make the requisite changes from cognition to behaviors, especially with regard to negative attitude. The enduring efficacy of CBT for MMT patients must be confirmed after treatment; we will follow the MMT patients in future studies. Third, the protocol of CBT may need to be revised and adapted in the future to address specific characteristics and factors for improving treatment retention for MMT.

## Conclusion

In sum, the findings of the present study support the efficacy of manual-based CBT in reducing opiate use and psychological stress and improving employment function for patients in MMT clinics. The long-term efficacy of CBT for reducing opiate use and improving psychosocial functions must be followed up after the end of treatment in the study. It is recommended that CBT can be combined with standard care of MMT treatment to improve treatment outcomes.

## Supporting Information

S1 CONSORT Checklist (DOC)

S1 Protocol (English Version) (DOCX)

S1 Protocol (Chinese Version) (DOC)
