# Supplementary material for: Efficacy of Cognitive Behavioral Therapy on Opiate Use and Retention in Methadone Maintenance Treatment in China: A Randomised Trial
Source: PLoS One. 2015 Jun 24;10(6):e0127598. doi: 10.1371/journal.pone.0127598 (PMC4479610; doi:10.1371/journal.pone.0127598)
Supplement: S1 Protocol (English Version) — (DOCX) [file pone.0127598.s002.docx]

Optimizing intervention for patients in community-based methadone maintenance treatment in China （protocol）

Organization: Shanghai Mental Health Center

Principal Investigator: Ph.D. Min Zhao

Project Director：Jiang Du

**Background**

Chronic drug use has been major public health concern for decades as it brought negative impacts on individual, family and society. Heroin has been being the mostly abused drug in China. Community-based methadone maintenance treatment (MMT) clinics have been built to cope with heroin dependence in China since 2004 and brought positive influences on helping Chinese heroin-dependents ^[1]^. There are some problems, such as low treatment retention and high dropout rate during MMT ^[2]^, which seriously impede the efficacy of MMT program.

**Study Objectives**

The objectives of the study are to evaluate efficacy of the optimizing intervention for Chinese heroin-dependents in MMT on reducing drug use and dropout rate, improving treatment retention and psychosocial functions; and to explore the relationships between drug use or treatment retention and psychosocial functions.

**Method and Study process**

**Participants and Study Procedure**

The study plans to recruit 240 heroin-dependents from four community-based MMT clinics located in Shanghai city through flyers and invitations. Participants will receive optimizing intervention or standard treatment in the first 26-week study period and all participants will be offered standard treatment in the second 26-week period. Eligible participants would be aged between 18 to 65 years old, heroin dependents diagnosed by clinicians with DSM-IV criteria. Participants would be excluded if they are schizophrenia patients with active psychosis symptoms and serious physical illness which prevent methadone medication. Eligible participants will be interviewed and assessed by trained investigators with instruments after informed consents are provided, then assigned randomly with computerized assignment procedure on the basis of stratification according to sex and methadone dose. All participants will be assessed at week 12, 26, 38 and 52. Randomized groups will be blind to assessors. Participants would be discharged from study and regarded as dropout from study if they do not attend MMT clinics for consecutive seven days.

**Instruments**

Participants will be assessed with the following instruments: self-designed questionnaire for collecting demographic characteristics, addiction severity index ^[3]^, the risk assessment battery ^[4]^; perceived stigma scale ^[5]^, self efficacy ^[6]^, self esteem inventory ^[7]^, social support ^[8]^, temperament and character inventory ^[9]^. Participants are also assessed with instruments of alcohol use disorders identification test ^[10]^, HIV-knowledge questionnaire ^[11]^, HCV knowledge questionnaire ^[12]^, methadone knowledge scale ^[13]^, Barratt impulsive scale ^[14]^, brief symptom inventory ^[15]^, Beck depression inventory ^[16]^, psychological stress scale ^[17]^; ways of coping questionnaire ^[18]^, which have been used in MMT clinics. All study data collected will be analyzed.

**Standard Care Treatment**

Participants in standard treatment group will receive daily medication of methadone and monthly health education during 26-week intervention period. The methadone dose will be increased gradually in the first week to a stable level which could control craving for heroin. Monthly health education consists of relapse prevention, self management, voluntary counselling and testing for HIV by both circulating pamphlets to participants and giving lectures.

**Optimizing Intervention Procedure**

Participants in the optimizing intervention group receive weekly individual session and monthly group session of CBT on the basis of standard treatment. Each individual session is approximately 45 minutes and one group session is approximately 90 minutes. The individual CBT sessions consist of signing treatment agreement, making individualised intervention protocol, coping-skills training, management of psychological stress and craving for drug use, balancing lifestyle. CBT group sessions consist of recognition and self-control of drug craving, harm reduction and relapse management. Optimizing intervention is developed and adapted to Chinese heroin-dependents on the basis of evidence-based intervention strategy through cooperation with experts of drug abuse treatment in integrated substance abuse program at UCLA. Specific intervention contents will be detailed in the CBT manual.

**Quality Control**

Psychotherapists and assessors in the study will be trained by superior investigators and rated pre- and post-training. The administration of study protocol and the psychological intervention will be supervised by one superior psychotherapist. Supervisions mainly in the form of case-based discussion will be taped and recorded and rated by another superior therapist.

**Outcome Measurements**

Treatment retention is calculated with percentage of participants still in treatment by end of study. Drug use is measured by both negative percentage of urine samples and self-reported drug use. Urine specimens will be collected biweekly for heroin and monthly for methamphetamine during assigned treatment except for baseline examination. Missing urine specimens are regarded as positive. The dropout rate is calculated as percentage of dropout participants with assigned sample by end of study. Improvements of participants are manifested with pre- and post- treatment changes in psychosocial function variables. The correlation between treatment retention and psychosocial function variables, and the relationship between urinalysis results and psychosocial function variables will be analyzed.

**Data Analyses**

Chi-square test and Kaplan-Meier survival analysis will be used to examine the differences of percentage of negative urine samples, treatment retention and dropout rate across conditions. An analysis of variance will be used to test differences of continuous variables, such as self-reported drug use and psychosocial function variables across conditions. Data of continuous variables from baseline to week 12, 26, 38 and 52 will be analyzed with general estimating equation. Regression analysis will be used to explore the relationship between treatment retention and psychosocial functions, drug use and psychosocial functions. Statistical tests are considered significant at p<.05.

**Reference**

1.Yin W, Hao Y, Sun X, Gong X, Li F, et al. (2010) Scaling up the national methadone maintenance treatment program in China: achievements and challenges. International journal of epidemiology 39: ii29-ii37.

2. Lu L, Zhao D, Bao Y-p, Shi J (2008) Methadone maintenance treatment of heroin abuse in China. The American journal of drug and alcohol abuse 34: 127-131.

3. McLellan AT, Luborsky L, Cacciola J, Griffith J, Evans F, et al. (1985) New data from the addiction severity index. Reliability and validity in three centers. J Nerv Ment Dis 173: 412–423.

4. Navaline HA, Snider EC, Petro CJ, Tobin D, Metzger D, et al. (1994) Preparations for AIDS vaccine trials. An automated version of the Risk Assessment Battery (RAB): enhancing the assessment of risk behaviors. AIDS Res Hum Retroviruses 10: S281–283.

# 5. Jason B. Luoma, Michael P. Twohig, Thomas Waltz, Steven C. Hayes, Nancy Roget, et al. (2007) An investigation of stigma in individuals receiving treatment for substance abuse. Addictive Behaviors 32: 1331–1346.

# 6. Sherer. M. & Maddux. JE (1982) The self-efficacy scale: Construction and validation. Psychological Reports 51: 663-671.

7. ARTHUR G. BEDEIAN, RALPH J, TEAGUE. JR, and ROBERT W. ZMUD (1977) TEST-RETEST RELIABILITY AND INTERNAL CONSISTENCY OF SHORT-FORM OF COOPERSMITH'S SELF-ESTEEM INVENTORY. Psychological Reports 41:1041-1042.

8. Guillermo Bernal, Mildred M. Maldonado-Molina, and María R. Scharrón del Río (2003) Development of a Brief Scale for Social Support: Reliability and validity in Puerto Rico. International Journal of Clinical and Health Psychology 3: 251-264.

9. De Fruyt F, Van De Wiele L, Van Heeringen C (2000) Cloninger's psychobiological model of temperament and character and the five-factor model of personality. Personality and individual differences 29: 441-452.

10. Saunders JB, Aasland OG, Babor TF, de la Fuente JR and Grant M (1993) Development of the Alcohol Use Disorders Identification Test (AUDIT): WHO collaborative project on early detection of persons with harmful alcohol consumption II. Addiction 88: 791-804.

11. Carey MP, Morrison-Beedy D, & Johnson BT (1997) The HIV-Knowledge Questionnaire: Development and evaluation of a reliable, valid, and practical self- administered questionnaire. AIDS and Behavior 1: 61-74.

12. Du, Jiang, Wang Zhen, Xie Bin, Zhao Min (2012) Hepatitis C Knowledge and Alcohol Consumption among Patients Receiving Methadone Maintenance Treatment in Shanghai, China. The American Journal of Drug and Alcohol Abuse 38: 228-232

13. Andrews S, Sorensen JL, Guydish J, Delucchi K, Greenberg B (2005) Knowledge and Attitudes About Methadone Maintenance Among Staff Working in a Therapeutic Community. J Maint Addict 3:47-59.

14. Patton JH, Stanford MS (1995) Factor structure of the Barratt impulsiveness scale. Journal of clinical psychology 51: 768-774.

15. Boulet J, Boss MW (1991) Reliability and validity of the Brief Symptom Inventory. Psychological Assessment: A Journal of Consulting and Clinical Psychology 3: 433-437.

16. Beck AT, Steer RA (1984) Internal consistencies of the original and revised Beck Depression Inventory. Journal of clinical psychology 40: 1365-1367.

17. Wang Z, Chen J, Boyd JE, Zhang H, Jia X, et al. (2011) Psychometric properties of the Chinese version of the Perceived Stress Scale in policewomen. PloS one 6: e28610.

18. Chan, David W (1994) The Chinese Ways of Coping Questionnaire: Assessing coping in secondary school teachers and students in Hong Kong. Psychological Assessment 6: 108-116.
