# Supplementary material for: Efficacy of Cognitive Behavioral Therapy on Opiate Use and Retention in Methadone Maintenance Treatment in China: A Randomised Trial
Source: PLoS One. 2015 Jun 24;10(6):e0127598. doi: 10.1371/journal.pone.0127598 (PMC4479610; doi:10.1371/journal.pone.0127598)
Supplement: S1 Protocol (Chinese Version) — (DOC) [file pone.0127598.s003.doc]

中国社区美沙酮维持治疗的优化干预方案研究（方案）

项目负责单位：上海精神卫生中心

项目负责人： 赵敏博士

项目主管： 杜 江

1. 背景

数十年来毒品依赖作为我国重要的公共卫生问题，给吸毒者本人、家庭和社会带来很大的负面影响。针对最常见的滥用毒品为海洛因，我国于2004年开始建立社区美沙酮维持治疗门诊并在帮助应对海洛因依赖问题方面确实起到了积极作用^【1】^。然而，美沙酮维持治疗模式中存在的诸多问题，如治疗依从性低，高脱失率^【2】^等严重阻碍了海洛因依赖者的康复。

1. 研究目标

本研究的目的是评价优化干预方案对提高美沙酮维持治疗患者的治疗依从性，减少毒品使用，降低脱失率，改善社会心理功能的疗效。

三、主要研究方法

**（一）研究对象及程序**

本研究拟分别从上海市四个社区美沙酮维持治疗门诊通过邀请、发放宣传手册等方式招募240例海洛因依赖患者。在前26周干预研究期间，研究对象分别接受优化心理干预和标准治疗，干预研究结束后，所有的研究对象均接受标准治疗。研究纳入标准为：年龄18-65岁之间；根据DSM-IV的诊断标准确诊为海洛因依赖。排除精神障碍发作期、因严重躯体疾病而影响美沙酮药物治疗者。在患者提供知情同意后由门诊医生对其进行评估，符合研究纳入标准的患者随后由接受过培训的研究人员对其进行访谈和问卷测评，随后按照以性别和美沙酮维持治疗剂量分层后制定的随机化方法对研究对象进行随机分组，并于12周、26周、38周及52周末对研究对象进行随访评估，参与评估的研究人员不知道研究对象的分组情况。研究对象如果连续7天未能参加美沙酮门诊治疗将会被要求退出研究并视为脱失病例。

**（二）研究工具**

研究所采用的调查问卷包括：自制问卷以收集个人信息、成瘾行为严重程度指数^【3】^、高危行为调查表^【4】^、病耻感调查表^【5】^、自我效能^【6】^、自尊^【7】^、社会支持调查表^【8】^、气质性格问卷^【9】^。因部分调查问卷，如酒精依赖筛查问卷^【10】^、HIV^【11】^、HCV知识问卷^【12】^、美沙酮维持治疗障碍调查表^【13】^、Barratt冲动行为调查表^【14】^、简明症状量表^【15】^、贝克抑郁问卷^【16】^、心理应激^【17】^和应对方式问卷^【18】^已经在门诊使用中，本研究将对所有问卷调查获得的数据进行综合分析。

**（三） 标准治疗**

分入美沙酮维持治疗组的研究对象每天服用美沙酮维持剂量的同时每月参加一次健康教育。在参加治疗的第一周，有初始剂量逐渐增加至稳定剂量，以能有效抑制渴求感。心理健康教育以讲座或者发放宣传册的形式指导患者如何预防复吸，自我管理和艾滋病自愿咨询和检测等内容。

**（四）优化干预措施**

分入优化干预组的研究对象在标准治疗的基础上，接受由治疗师提供每周一次个体认知行为干预和每月一次团体心理干预。个体心理治疗和团体心理治疗分别约为45分钟和90分钟，个体心理治疗内容包括签订治疗协议，制定个体化治疗方案，应对技能训练，心理应激和渴求管理，平衡生活方式等。团体干预的内容包括渴求识别和自我控制，减低伤害，复吸管理等。心理干预手册是由研究团队成员与美国洛杉矶大学加利福尼亚分校的药物滥用综合治疗项目组专家合作，根据国际上具有循证医学基础的，编写及制定适合我国海洛因成瘾患者的优化干预措施，详细干预措施有单独的治疗手册。

**四、质量控制**

执行评估和心理干预的研究人员均接受相应的培训及培训前后的评估，由一名高级研究人员对研究方案和干预措施的执行情况进行监督和督导，督导的形式主要为案例讨论，记录督导内容并录音，由另一名高级治疗师对督导内容进行讨论和评估。

**五、评价指标**

治疗依从性以截止治疗结束时，完成全部治疗的人数与随机分组的研究对象之比计算。研究对象毒品滥用情况分别以尿检阴性率和自我报告来评估。研究对象于入组时进行海洛因、冰毒检查后，以后每2周1次进行尿海洛因和每个月1次尿冰毒检测，患者未提供尿检样本则视为阳性。脱失率是以在研究期间脱失，未完成治疗的患者与随机分组的研究对象之比计算。研究对象在社会心理功能方面的改变可表现为治疗前后的差异。研究也将分析研究对象的治疗依从性与社会心理变量间的相关性，尿检结果与社会心理功能变量间的相关关系。

**六、统计分析**

本研究以卡方检验方法分析如尿检阴性率、治疗依从性、脱失率等计数资料的组间差异。以方差分析计算连续性变量，如自我报告的药物使用天数，社会心理功能变量的组间差异。本研究将采用广义估计方程分析连续性变量结果从基线至12周末，26周末，38周末和52周末的变化。以回归方法分析治疗依从性与社会心理学变量，毒品滥用与社会心理变量间的相关关系。所有统计指标均以P值小于0.05为具有显著性统计学意义。

**参考文献**

1.Yin W, Hao Y, Sun X, Gong X, Li F, et al. (2010) Scaling up the national methadone maintenance treatment program in China: achievements and challenges. International journal of epidemiology 39: ii29-ii37.

2. Lu L, Zhao D, Bao Y-p, Shi J (2008) Methadone maintenance treatment of heroin abuse in China. The American journal of drug and alcohol abuse 34: 127-131.

3. McLellan AT, Luborsky L, Cacciola J, Griffith J, Evans F, et al. (1985) New data from the addiction severity index. Reliability and validity in three centers. J Nerv Ment Dis 173: 412–423.

4. Navaline HA, Snider EC, Petro CJ, Tobin D, Metzger D, et al. (1994) Preparations for AIDS vaccine trials. An automated version of the Risk Assessment Battery (RAB): enhancing the assessment of risk behaviors. AIDS Res Hum Retroviruses 10: S281–283.

# 5. Jason B. Luoma, Michael P. Twohig, Thomas Waltz, Steven C. Hayes, Nancy Roget, et al. (2007) An investigation of stigma in individuals receiving treatment for substance abuse. Addictive Behaviors 32: 1331–1346.

# 6. Sherer. M. & Maddux. JE (1982) The self-efficacy scale: Construction and validation. Psychological Reports 51: 663-671.

7. ARTHUR G. BEDEIAN, RALPH J, TEAGUE. JR, and ROBERT W. ZMUD (1977) TEST-RETEST RELIABILITY AND INTERNAL CONSISTENCY OF SHORT-FORM OF COOPERSMITH'S SELF-ESTEEM INVENTORY. Psychological Reports 41:1041-1042.

8. Guillermo Bernal, Mildred M. Maldonado-Molina, and María R. Scharrón del Río (2003) Development of a Brief Scale for Social Support: Reliability and validity in Puerto Rico. International Journal of Clinical and Health Psychology 3: 251-264.

9. De Fruyt F, Van De Wiele L, Van Heeringen C (2000) Cloninger's psychobiological model of temperament and character and the five-factor model of personality. Personality and individual differences 29: 441-452.

10. Saunders JB, Aasland OG, Babor TF, de la Fuente JR and Grant M (1993) Development of the Alcohol Use Disorders Identification Test (AUDIT): WHO collaborative project on early detection of persons with harmful alcohol consumption II. Addiction 88: 791-804.

11. Carey MP, Morrison-Beedy D, & Johnson BT (1997) The HIV-Knowledge Questionnaire: Development and evaluation of a reliable, valid, and practical self- administered questionnaire. AIDS and Behavior 1: 61-74.

12. Du, Jiang, Wang Zhen, Xie Bin, Zhao Min (2012) Hepatitis C Knowledge and Alcohol Consumption among Patients Receiving Methadone Maintenance Treatment in Shanghai, China. [The American Journal of Drug and Alcohol Abuse](http://chinesesites.library.ingentaconnect.com/content/apl/lada;jsessionid=62tokefhni26f.victoria) 38: 228-232

13. Andrews S, Sorensen JL, Guydish J, Delucchi K, Greenberg B (2005) Knowledge and Attitudes About Methadone Maintenance Among Staff Working in a Therapeutic Community. [J Maint Addict](http://www.ncbi.nlm.nih.gov/pubmed/23525520) 3:47-59.

14. Patton JH, Stanford MS (1995) Factor structure of the Barratt impulsiveness scale. Journal of clinical psychology 51: 768-774.

15. Boulet J, Boss MW (1991) Reliability and validity of the Brief Symptom Inventory. Psychological Assessment: A Journal of Consulting and Clinical Psychology 3: 433-437.

16. Beck AT, Steer RA (1984) Internal consistencies of the original and revised Beck Depression Inventory. Journal of clinical psychology 40: 1365-1367.

17. Wang Z, Chen J, Boyd JE, Zhang H, Jia X, et al. (2011) Psychometric properties of the Chinese version of the Perceived Stress Scale in policewomen. PloS one 6: e28610.

18. Chan, David W (1994) The Chinese Ways of Coping Questionnaire: Assessing coping in secondary school teachers and students in Hong Kong. Psychological Assessment 6: 108-116.
